# miRNA Delivery by Nanosystems: State of the Art and Perspectives

**DOI:** 10.3390/pharmaceutics13111901

**Published:** 2021-11-09

**Authors:** Fernanda C. Moraes, Chantal Pichon, Didier Letourneur, Frédéric Chaubet

**Affiliations:** 1 LVTS, INSERM U1148, Université Sorbonne Paris Nord, Université de Paris, 75018 Paris, France; fer_nanda103@hotmail.com (F.C.M.); frederic.chaubet@univ-paris13.fr (F.C.); 2 Centre de Biophysique Moléculaire, CNRS UPR4301, Université d’Orléans, 45071 Orléans, France; chantal.pichon@cnrs.fr

**Keywords:** miRNA delivery, non-viral vectors, nanoparticles

## Abstract

MicroRNAs (miRNAs) are short (~21–23 nucleotides), non-coding endogenous RNA molecules that modulate gene expression at the post-transcriptional level via the endogenous RNA interference machinery of the cell. They have emerged as potential biopharmaceuticals candidates for the treatment of various diseases, including cancer, cardiovascular and metabolic diseases. However, in order to advance miRNAs therapeutics into clinical settings, their delivery remains a major challenge. Different types of vectors have been investigated to allow the delivery of miRNA in the diseased tissue. In particular, non-viral delivery systems have shown important advantages such as versatility, low cost, easy fabrication and low immunogenicity. Here, we present a general overview of the main types of non-viral vectors developed for miRNA delivery, with their advantages, limitations and future perspectives.

## 1. Introduction

Nowadays, the development of innovative therapies based on nucleic acids (including plasmid DNA, antisense oligonucleotides, messenger RNAs, siRNAs, miRNAs, etc.) and proteins (monoclonal antibodies, growth factors, hormones, therapeutic enzymes, synthetic oligopeptides, etc.) has emerged as highly specific pharmaceutical agents [1,2]. Gene therapy uses nucleic acids for the treatment, cure or prevention of human disorders and has gained considerable attention over the past three decades as it holds great promise for the treatment of many diseases such as cancers, genetic diseases, viral infections and cardiovascular disorders [3,4].

RNA interference (RNAi) is a well-known post-transcriptional endogenous pathway for gene expression silencing. RNAi is a regulatory mechanism that uses double-stranded RNA (dsRNA) to specific gene silencing by targeting mRNA for degradation. It has been widely used to study the cellular function of genes. The most known and exploited molecules are small interfering RNAs (siRNAs) and microRNAs (miRNAs) with 20–25 nt and 20–23 nt of length, respectively [5,6,7,8,9,10,11]. They have emerged as future medicines due to their multiple unique features and advantages when compared to DNA-based therapy [12,13]. RNA therapeutics do not have the risk of genomic integration, do not need to cross the nucleus membrane, and are expressed instantaneously [14,15]. Research indicates that small RNAs are involved in relatively cell generic functions being implicated in the control of many fundamental cellular and physiological processes such as cellular differentiation, metabolism, proliferation, cell cycle control, migration and apoptosis [16].

The first therapeutic small interfering RNA was approved by the Food and Drug Administration (FDA) in 2018, Onpattro^®^ (patisiran), a lipid complex injection to treat hereditary polyneuropathy of hereditary transthyretin-mediated amyloidosis (hATTR) in adults [17].

Compared with siRNAs, miRNAs have a larger therapeutic application once they are able to affect multiple pathways/cellular processes rather than specific targets because miRNA recognition requires the binding to a much shorter seed sequence (2–8 nt) instead of the entire nucleotide sequence of a siRNA [18,19,20]. miRNAs modulate gene expression by imperfectly binding to targeted messenger RNAs (mRNA), which results in mRNA degradation or translational repression [21]. At the target, miRNAs can generate strong, sustained and comprehensive biological effects through endogenous gene silencing at nanomolar concentrations [22,23].

MicroRNAs-based therapeutics have been studied to treat various diseases including cancer, cardiovascular pathologies, diabetes and neuroinflammation [24,25]. Hence, the ability to control the expression of in vivo miRNA will serve as the basis for the development of treatments. Two different therapeutic approaches are generally used for miRNA-based therapeutics: miRNA inhibition and miRNA replacement. miRNA inhibition is used in situations when the target miRNA is overexpressed. It involves the use of synthetic single-stranded RNAs, called antagomirs and miRNA sponges, that are partially or fully complementary to the target miRNA and act as miRNA antagonists (anti-miRNA) by blocking the binding to endogenous mRNA targets [26,27]. For example, several miRNAs showed to be involved with important cancer pathways (cell migration, proliferation, apoptosis) and thus the inhibition of those overexpressed miRNAs would be helpful to control disease evolution [5,10,28,29]. On the other hand, miRNA mimics (replacement therapy) is employed when the target miRNA is down-regulated. This replacement could be obtained as well with the use of small synthetic double-stranded molecules processed into functional miRNA or miRNA expression vectors to induce expression of a miRNA in cells and the delivery of the miRNA itself. The goal of those strategies is to restore miRNA levels using synthetic double-stranded miRNAs to mimic the function of the target miRNAs and to bind specifically to its target mRNA to produce post-transcriptional repression of the gene [30,31,32].

Despite the emergence of miRNA therapeutics, they have not been yet translated into FDA-approved drugs, but some candidates are in clinical development or in phase 1 and phase 2 of clinical trials [33]. Chakraborty et al. (2020) listed the major miRNA based therapeutics that are being evaluated in clinical trials [34]. At present, there are several biotech companies working exclusively on the development of miRNA therapeutics, such as Regulus Therapeutics, Miragen and MiRNA Therapeutics.

Currently, the main obstacle preventing the implementation of miRNA-based therapies in clinical practice is the lack of an efficient delivery system, which is also the case of other nucleic acids therapeutics. Despite their small size, miRNAs are different from conventional drugs in that they cannot passively diffuse across lipid membranes into target cells and exhibit very limited biological stability [35]. Therefore, a major challenge for gene therapy is the development of efficient nucleic acid delivery systems to target cells, with minimal toxicity and high bioavailability [36]. Combining the use of miRNAs with nanotechnology is a promising strategy for the development of clinically viable treatment options. In this review, we provide a general overview of the different classes of non-viral carriers developed for miRNA delivery, their advantages and limitations, their therapeutic applications, as well as future perspectives in the design and development of miRNA delivery technologies.

## 2. Delivery Vehicles for miRNA Therapeutics

Vectors employed for gene delivery purposes can be divided into two main groups: (a) viral carriers, where the genetic material is incorporated into a virus, and (b) non-viral carriers, including cationic molecular carriers such as lipids and polymers, which can form electrostatic interactions with nucleic acids for the gene delivery to cells [4].

**Figure 1:**
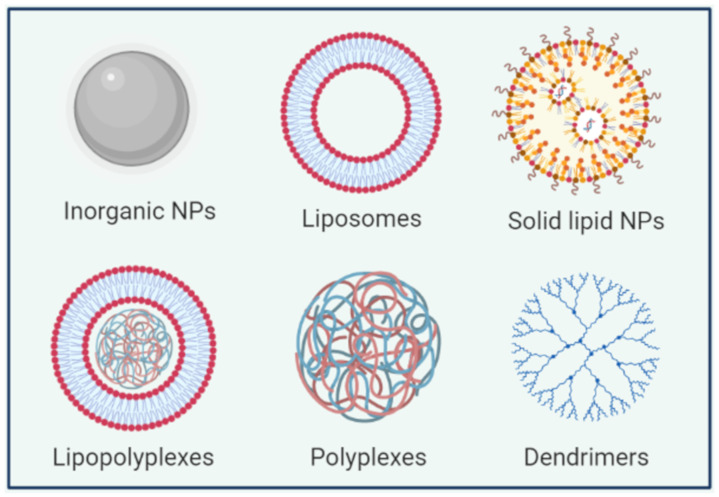
A schematic representation of some nanocarriers used for miRNA delivery.

The majority of approved vectors for gene therapy currently applied in clinical protocols are viral vectors due to their high transfection efficiency [37]. Viruses such as retroviruses, lentiviruses (as HIV), adenoviruses and adeno-associated viruses can be diverted as gene delivery systems by the replacement of part of viral genome with a therapeutic gene [38]. However, limitations of viral vector delivery systems such as immunogenicity and mutagenicity could restrict their clinical application [4,39,40]. These limitations have prompted the development of non-viral gene carriers using different materials (lipid-based nanoparticles (NPs), polymer-based carriers and inorganic NPs) (Figure 1). Those non-viral carriers have considerable advantages compared to viral vectors such as relatively low immunogenicity, low cost and versatility. Those carriers can accommodate nucleic acids with large size and they can be more easily functionalized with specific ligands allowing their targeting to a given organ or cell type. The choice of the appropriate carrier is influenced by the route of administration and therapeutic objectives [41].

### 2.1. Inorganic Nanoparticles

Synthetic materials present important advantages as vehicles for miRNA delivery due to the possibility to produce structures with tunable size, morphology and controlled composition and their simplified manufacturing procedures [42,43]. Depending on the nature of the material core, different strategies can be used for nucleic acid incorporation: encapsulation within the material matrix, adsorption with materials bearing cationic moieties or covalent attachment when the NP surface can be modulated by reactive groups [44,45,46,47]. Inorganic materials that have been proposed for the intracellular delivery of miRNAs includes examples such as gold, silica and iron oxide nanoparticles (Table 1) [47,48].

Owing to their mechanical and chemical stability, gold nanoparticles have received great attention as non-viral gene delivery system [31]. Gold NPs can be easily functionalized with thiol or amino groups to allow surface loading of negatively charged miRNAs through electrostatic interaction [49,50]. Sukumar et al. developed polyfunctional gold-iron oxide nanoparticle (polyGION) for intranasal delivery of antimiR-21 and miR-100. They functionalized the surface of polyGION NPs with chitosan cyclodextrin (CD-CS) hybrid polymers. Upon the inclusion of CD-CS coating, the surface potential of these NPs shifted from −15 mV to +39 mV owing to excess of positively charged free amine groups of chitosan. A loading efficiency of almost 80% of added miRNA was achieved [47]. However, the potential cytotoxicity of the NPs could limit their clinical application. For example, gold NPs 5 nm in diameter disrupt the cytoskeletal organization of fibroblasts after 72 h of exposure [51].

Silica has also been used for miRNA delivery because of its biodegradability and easy functionalization [31,52]. For example, silica based-NPs have been developed for the delivery of miR-34a, which is a part of a tumor-suppressor miRNA family often down-regulated in cancers. Tivnan et al. developed functionalized disialoganglioside GD2-antibody conjugated porous silica nanoparticles for the targeted delivery of miR-34a to neuroblastoma tumors. GD2 is a glycolipid overexpressed on the surface of neuroblastoma tumor cells. Nanoparticles encapsulating miR-34a resulted in significantly decrease of tumor growth and increased apoptosis thanks to the antibody-mediated cell cytotoxicity [44]. Silica dioxide nanoparticles (SiO_2_-NPs) were also effectively used for the delivery of miR-34a into normal and cancer epithelial cells grown in vitro. The miRNA delivery using SiO_2_-NPs resulted in reduction of mammary tumor growth. Interestingly, miR-34a-based SiO_2_-NPs delivery was equally effective as by lipofection, with the advantage of being more suitable for clinical applications [53].

One benefit of using inorganic nanoparticles is the possibility of applying them for theranostic purposes. Indeed, in addition of their capacity to deliver nucleic acids, they are also suitable for magnetic resonance imaging (MRI). Leder et al. developed silica-based micron-sized iron oxide-containing particles (sMPIO) for the delivery of miRNA and MRI-based tracking of transplanted cells thanks to magnetic properties of the iron oxide core. The oligonucleotide was covalently linked on the NPs surface, assuring higher stability and efficient delivery [48]. Similarly, Yang et al. developed a poly(amidoamine) dendrimer-grafted gadolinium-functionalized nanographene oxide (Gd-NGO) nanoparticles for combined miRNA delivery and MRI [54].

**Table 1:** Inorganic-based miRNA delivery systems.

Delivery System	miRNA	Therapeutic Application	Particle Size	Refs.
Gold-iron oxide NPs	anti-miR-21 miR-100	Glioblastoma	10–50 nm	[47]
Gold NPs	miR-21	Cancer	61.3 nm	[55]
Gold NPs	miR-34a miR-200a let7-a	Cancer	2 nm	[56]
Silica NPs	miR-34a	Neuroblastoma	N/A	[44]
Silica dioxide NPs	miR-34a	Breast cancer	12–18 nm	[53]

N/A: not applicable.

### 2.2. Lipid-Based Nanoparticles

Lipid-based nanoparticles (LNPs) represent one of the most widely used strategies for in vivo delivery of small interfering RNAs [57,58]. Several classes have been studied for miRNA delivery, including liposomes, solid lipid nanoparticles and hybrid lipid-polymer NPs (Table 2).

Liposomes are drug delivery systems composed of a lipid bilayer that contains the miRNA encapsulated in the hydrophilic aqueous core or complexed with the lipids on the surface [49,59]. In order to achieve high nucleic acid loading, cationic lipids are generally preferable because they can easily associate with negatively charged nucleic acids to form complexes named lipoplexes [60]. Among such cationic lipids, quaternary amine based cationic lipids, such as N-[1-(2,3-dioleyloxy) propyl-N,N,N-trimethylammonium chloride (DOTMA) and 1,2-dioleoyl-3-trimethylammonium-propane (DOTAP), are currently used due to their permanent positive charge [61]. Lipoplexes prepared with cationic lipids containing LNPs, such as the lipofectamine reagent, have shown to be useful for in vitro transfection [62]. However, in vivo applications of lipoplexes could be limited by the high toxicity associated with most of permanently charged cationic lipids.

In order to avoid the toxicity issues, LNPs have been developed from ionizable cationic lipids with primary, secondary, or tertiary amines in the headgroup and apparent pKa values below 7. These LNPs could be loaded with nucleic acid at pH values below the pKa of the ionizable lipid where it is positively charged while exhibiting relatively neutral surface under physiological conditions [41,57,63,64]. Yung et al. prepared LNPs based on cationic lipids bearing tertiary and quaternary amine groups for therapeutic delivery of antimiR-21. The design combined the advantage of the high charge density provided by the permanent positive charge of quaternary ammonium groups and the pH responsiveness behavior of tertiary amines in order to obtain a balanced charge-vs-pH release profile for efficient miRNA delivery [65].

LNPs may also have additional components in their formulation such as cholesterol (Chol) and dioleoylphosphatidyl ethanolamine (DOPE), known as “helper lipids” which contribute to increase nanoparticle stability and reduce their toxicity. Moreover, the incorporation of polyethylene glycol (PEG) is frequently employed to reduce NPs aggregation in the presence of serum proteins and increase circulation time [49,59]. In the study of Campani et al., they investigated the use of different cationic lipids, neutral lipids, and PEGylated lipids to achieve optimal miRNA delivery. By combining different lipids, they developed formulations with good technological characteristics and stable under physiological conditions with a low hemolytic profile. The in vitro uptake studies on glioblastoma cell lines showed that the developed nanosystems allowed higher miRNA delivery when compared to commercial lipofectamine reagent [58].

Trang et al. explored the potential of a neutral lipid emulsion (NLE) for in vivo delivery of miR-34a mimics. They demonstrated efficient therapeutic delivery of miRNA formulations with reduction in tumor size. Particles formulated with neutral lipids are less susceptible to aggregation in biological fluids, to be filtered by the liver, or be taken up by scavenging macrophages [66]. In the work of Nogueira et al. small interfering RNAs were encapsulated in the aqueous core of DOPE-derived neutral PEGylated liposomes by the hydration of the lipid film with a concentrated solution containing the payload prior to liposome formation. A loading efficiency around 100% was achieved using this strategy. The encapsulation of small RNAs inside the hydrophilic core of liposomes can avoid premature payload release in the presence of biological fluids [67].

Solid lipid nanoparticles (SLNs) are another class of lipid-based nanoparticles that have been developed for miRNA delivery. When compared with liposomes, they have a solid lipid core, which makes difficult the encapsulation of anionic miRNA. The use of cationic lipids can be an alternative to overcome this drawback [68]. Notably, SLNs are able to load hydrophilic and lipophilic active substances better than other colloidal systems. For example, SLNs were used to co-deliver anti-miR-21 and pemetrexed to glioblastoma cells. Anti-miR-21 and pemetrexed were effectively encapsulated into SLNs (over 90%). The SLNs allowed a significantly higher cellular uptake of pemetrexed by glioblastoma cells when compared to a free drug [69]. 

Similarly, in another study SLNs were employed for the co-delivery of miR-34a, a cancer-specific anti-oncogene and paclitaxel. For this purpose, paclitaxel was encapsulated into SLNs first, and then miR-34a was self-assembled by electrostatic interaction. The cationic lipid dimethyldioctadecylammonium bromide (DDAB) was used for complex miR-34a. High rates of encapsulation were obtained for both paclitaxel (93.05%) and miRNA (95.13%). The co-deliver of miR-34a and paclitaxel can enhance anti-cancer therapy by exploiting the synergistic effect of the two drugs [70].

Hybrid lipid-polymer NPs termed also as lipopolyplexes (LPR or LPP) are another type of NPs suitable for miRNA delivery. Those nanoparticles comprise nucleic acids complexed with biodegradable polymer coated/encapsulated with a lipid bilayer. The nucleic acids are protected via the complexation with polymer and the liposomal surface allows an easy targeting with different ligands and substitution with stealth moiety for long half-life in the blood [71]. Huang et al. have proposed one type of lipopolyplexes made with polyethylenimine (PEI) and DOPE/linoleic acid/DMG-PEG on which transferrin (Tf) molecules were post-inserted. Those lipopolyplexes delivered miR-29b in acute myeloid leukemia (AML) cells. Mice engrafted with AML cells treated with Tf-NP-miR-29b had significantly longer survival compared with Tf-NP-scramble or free miR-29b [72]. The same formulation was proposed for the delivery of miR-1 transferrin-mediated NP delivery and resulted in a 3-fold higher delivery efficiency compared to NP without transferrin modification. Tf-NP-miR-1 treatment on glioblastoma multiforme (GBM) spheres significantly reduced the migration of GBM spheres by 30–50%, highlighting the potential of these systems for GBM treatment [73]. Histidylated lipopolyplexes are also one good representative of hybrid lipid-polymer-based NPs. The imidazole group present on both polymer and liposomes confer them an acid-mediated membrane destabilization of endosomes due to membrane fusion and/or a proton sponge mechanism favoring nucleic acid delivery in the cytosol. Those lipopolyplexes are versatile as they are able to deliver efficiently pDNA, siRNA and miRNA in different cell types [74,75,76,77,78]. Recently, Simion et al., reported the efficacy and intracellular trafficking of miRNA lipopolyplexes made with a miRNA complexed with a ionizable histidinylated linear PEI polymer and ionizable liposomes made with O,O-dioleyl-*N*-[3 *N*-(*N*-methylimidazolium iodide) propylene] phosphoramidate and the O,O-dioleyl-*N*-histamine phosphoramidate. Those NPs have a mean size around 117.9 ± 5.4 nm and a global charge of +49.1 ± 4.3 mV. Their delivery efficiency in U87 MG glioblastoma cells was higher to that of RNAimax, a well-used gold standard. Interestingly, they were successfully used for in vivo miRNA delivery in the brain when infused via a convection-enhanced method for a locoregional delivery [79]. It worth noting that lipopolyplexes based-formulation is amongst the different panel of NPs proposed for COVID-19 mRNA based vaccine by Stemirna Therapeutics [80].

**Table 2:** Lipid-based miRNA delivery systems.

Delivery System	miRNA	Therapeutic Application	Lipids Used	Particle Size	Refs.
Liposomes/ Lipoplexes	anti-miR-21	Lung cancer	DODMA/DOTAP/ DOPC/CHOL/mPEG-DPPE	150 nm	[65]
	miR-603	Glioblastoma	DOTAP/DOTMA/ DC-CHOL	120–160 nm	[58]
	miR-29b	Lung cancer	DOTMA	84 nm	[81]
	miR-133a	Glioblastoma	KLN25/MM27	180.9 nm	[79]
	miR-499	Cancer	DPPC/DOPE/ CHOL/DCP-TEPA	200 nm	[82]
	miR-101	Acute myeloid leukemia	DPPC/DOTAP/CHOL/ mPEG2000-DSPE	126.6 nm	[83]
	miR-101	Hepatocellular carcinoma	DOTAP	150 nm	[84]
SLNs	anti-miR-21	Glioblastoma	DDAB/tristearin/glyceryl tripalmitate/1-α-Phosphatidylcholine	124.9 nm	[69]
	miR-34a	Lung cancer	DDAB/Glyceryl monostearate/Soy phosphatidylcholine/CHOL	220 nm	[70]
LPP	miR-29b	Acute myeloid leukemia	DOPE/linoleic acid/DMG-PEG	137–147 nm	[72]
	miR-133a	Glioblastoma	KLN25/MM27	117.9 nm	[79]

Abbreviations: DODMA: 1,2-dioleyloxy-3-dimethylaminopropane; DOTAP:1,2-dioleoy-1-3- trimethylamonium propane; DOPC: 1,2-dioleoyl-sn-glycero-3-phosphatidylcholine; CHOL: cholesterol; DPPE: 1,2-dipalmitoyl-sn-glycero-3-phosphoethanolamine; DOTMA: *N*-[1-(2,3-dioleyloxy) propyl-*N*,*N*,*N*-trimethylammonium chloride; DC-CHOL: 3β-[*N*-(Dimethylaminoethane)carbamoyl]cholesterol; DMG-PEG: 1,2-dimyristoylsn-glycerol, methoxypolyethylene glycol KLN25: O,O-dioleyl-*N*-[3 *N*-(N-methylimidazolium iodide) propylene] phosphoramidate; MM27: O,O-dioleyl-*N*-histamine phosphoramidate; DOPE: dioleoylphosphatidyl ethanolamine; DCP-TEPA: dicetyl phosphate-tetraethylenepentamine; DPPC: dipalmitoylphosphatidylcholine; mPEG2000-DSPE: monomethoxy polyethylene glycol 2000-distearoyl phosphatidylethanolamine; DDAB: dimethyldioctadecylammonium bromide.

### 2.3. Polymeric Nanoparticles

Three main types of polymeric nanocarriers have been studied for miRNA delivery and are represented by polyplexes, polylactic-*co*-glycolic acid (PLGA) NPs and dendrimers (Table 3). The miRNA can be incorporated to the delivery systems by complexation (electrostatic interactions), conjugation (covalent linkers), or encapsulation [18,85].

Polyethyleneimine (PEI), a positively charged linear PEI (lPEI) or branched PEI (bPEI), is the most studied polymer being considered the “gold standard” for gene transfection protocols [13]. PEI consists of multiple positively charged amines (primary, secondary and tertiary amino groups) depending on the linear or branched structure of the polymer. Its advantages are a high cationic charge density and buffering capacity due to the protonation of primary amines that enables endosomal escape through the “proton sponge effect” in which a change in the osmolarity of the acidic vesicles results in endosomal swelling and rupture allowing nucleic acid release [18]. Radmanesh et al. used deoxycholic acid modified PEI for miR-210 delivery. They demonstrated an efficient balance between polyplexes stabilization and cargo release into the cytosol [86]. In the work of Gao et al., a polyethylene-poly l-Lysine (PEI-PLL) derivative was successfully synthesized and confirmed to transfect plasmid and miRNA more effectively than PEI in MCF-7 cells (human breast cancer cells) [87]. Nevertheless, the lack of degradability of PEI with a risk to accumulate in the body, in particular after repeated administration, largely limited their use [88]. In vivo application of PEI was shown to be limited by a molecular weight-dependent cytotoxicity related with an excess of positive charges which leads to non-specific interactions and aggregation in the bloodstream, while polymers based on amino acids such as poly(lysine) are known to be immunogenic [4,89,90].

Alternatively, PLGA has been frequently investigated to formulate NPs for the delivery of miRNA [85,91,92]. The slow degradation profile of PLGA is ideal for the controlled release of genes [93]. Although the biocompatibility of PLGA, its hydrophobicity and rapid opsonization in circulation are the main limitations for their use for miRNA delivery. To facilitate miRNA loading, many PLGA NPs are typically prepared with the incorporation of additional positively charged polymers. In the work of Kapadia et al., they synthetized layer-by-layer assembled nanoparticles (LbL NPs) comprised of spherical biodegradable PLGA core surrounded by alternating layers of PLL and miR-34a. By combining both polymers, they reduced the risk of systemic toxicity. Moreover, LbL NPs prepared from PLGA cores offer the possibility for co-drug delivery by loading other therapeutic agents into the PLGA core [85]. For efficient encapsulation (~78%) of miR-150 mimics in PLGA nanoparticles, Singh et al., incorporated PEI to the formulation of PLGA NPs using double emulsion solvent evaporation method [92]. Devulapally et al. developed PLGA-b-PEG-NPs loaded with antisense-miRNAs using water-in-oil-in-water (*w*/*o*/*w*) double emulsion method for breast cancer therapy. The PEGylation of PLGA can increase the circulation time of NPs [91].

Another class that has been investigated for miRNA delivery are dendrimers. They are a class of synthetic macromolecules characterized by tree-like structures with a high density of cationic charges. The high structural flexibility of dendrimers allows a more efficient interaction with nucleic acids and the unique dendritic architecture enable the design of advanced drug delivery systems with controlled monodispersity and surface functionality [94,95,96]. Moreover, by changing the composition of the different parts of dendrimers (core, surface and generation) it is possible to engineer NPs with different biodistribution profiles [97]. There are different types of dendrimers such as poly (propylene imine) (PPI) dendrimers, poly(l-lysine) dendrimers, polyamidoamine (PAMAM) dendrimers, etc. PAMAM, a highly branched spherical polymer, is the most widely studied. PAMAM dendrimer can be synthesized, tailored and characterized for any one or multifunctional group on its surface such as amine, carboxylate, hydroxyl, etc., and tertiary amine groups in the core. The presence of tertiary amine groups can promote miRNA intracellular release via the proton sponge effect [95,98]. Few works have used PAMAM for miRNA delivery. Sayed et al. designed poly (amidoamine)-histidine (PAMAM-His) nanocarriers for the delivery of miRNA in injured cardiomyocytes. The conjugation of PAMAM with histidine resulted in the reduction of polymer cytotoxicity and increased buffering capacity. At the highest tested concentration (150 μg/mL), PAMAM-His showed approximately 80% cell viability while commercial lipofectamine treatment resulted in approximately 61% of cell viability. PAMAM-His NPs effectively delivered miRNAs to the cardiomyocytes and prevented the hypoxia/reperfusion-induced apoptosis after myocardial infarcts [99]. In another study, PAMAM dendrimer was employed as a carrier to co-deliver anti-miR-21 and 5-fluorouracil (5-FU) to glioblastoma cells. PAMAM was simultaneously loaded with 5-FU and anti-miR-21 to form NPs smaller than 100 nm. The unique structure of PAMAM allowed them to interact with miRNA through charge-based interactions and facilitated the entrapment of chemotherapeutics in the interior of PAMAM through hydrophobic interactions [100].

Besides the use of synthetic polymers, several natural carbohydrate polymers have been proposed due to their low toxicity, low immunogenicity and generally good hemocompatibility properties [39,101]. Chitosan is the only naturally existing cationic polysaccharide and has received the most attention as gene delivery vehicle due to its high positive charge density [40,102]. The glucosamine unit in chitosan backbone provides primary amine groups which is positive charged at acidic pH conferring the ability to form complexes with polyanions [103]. Chitosan-based carriers have been largely used for the delivery of plasmid DNA (pDNA) [37,104], siRNA [105,106] and miRNA [107].

Santos-Carballal et al. studied the physicochemical and biological properties of chitosan polyplexes with miR-145. They found that ideal complexes were formed using chitosans with a molecular weight of ~40 kDa, and a N/P ratio of 1.5, resulting in transfection efficiencies similar to the commercial reagent used as positive control [108]. Kaban et al. used chitosan as carrier for the delivery of miRNA to breast cancer cell lines. Full complexation between chitosan and miRNA was obtained at a N/P ratio of 4 [109]. Cosco et al. developed nanocomplexes with a combination of chitosan and PLGA for miR-34a delivery. Chitosan was added in order to provide a positive surface charge onto PLGA nanoparticles allowing miRNA complexation. Polyplexes made with chitosan allowed a high level of entrapment efficiency of the miR-34a along with a high degree of transfection and a significant in vitro antitumor effect against multiple myeloma cells [110]. However, in spite of its interesting profile, chitosan has its own drawbacks such as low water solubility at physiological pH because of partial protonation of primary amino groups, which can affects nucleic acid complexation, and stability of complexes in blood [111,112].

Hyaluronic acid (HA) has been also used to prepare miRNA polyplexes. It is a natural anionic polysaccharide that can be used due to their targeting properties. For example, Deng et al. co-encapsulated miR-34a and doxorubicin into nanocomplexes composed of chitosan and hyaluronic acid prepared through a facile ionotropic gelation method in water. HA endowed tumor-targeting properties through specifically binding to CD44 molecule, an integral membrane glycoprotein over-expressed on the surface of various tumor cells. Complexes exhibit a good serum stability protecting miRNA from degradation, characteristic suitable for in vivo application [113]. In another study, HA was used to construct innovative nanocapsules based on polyelectrolyte complexes (PECs) between HA and protamine sulfate (PS). In vitro and in vivo experiments illustrated high miR-34a encapsulation efficiency (more than 90%) into HA-PS complexes and deliver into breast cancer cells or breast cancer tissues [114].

**Table 3:** Polymeric-based miRNA delivery systems.

Delivery System	miRNA	Therapeutic Application	Polymers	Particle Size	Refs.
Polyplexes	miR-210	Ischemic heart disease	PEI-Deoxycholic acid (DA)	100–180 nm	[86]
	anti-miR 21	Breast cancer	PEI-PLL	300 nm	[87]
	miR-34a	Prostate tumor	Chitosan	N/A	[107]
	miR-34a	Breast cancer	Chitosan/ Hyaluronic acid	185–214 nm	[113]
	miR-34a	Breast cancer	Hyaluronic acid/ Protamine sulfate	201 nm	[114]
	miR-124	Neurodegenerative disorders	Chitosan	222 nm	[115]
	miR-145	Breast cancer	Chitosan	190 nm	[108]
	miR-145	Breast cancer	Chitosan/Carboxymethyl dextran (CMD)	30–695 nm	[116]
	miR-181a	Chronic myeloid leukemia	Pullulan spermine (PS)	200–250 nm	[117]
	miR-200 miR 141	Breast cancer	Chitosan	296–380 nm	[109]
	miR-126	Angiogenesis	Trimethyl (TMC) chitosan	98–342 nm	[118]
PLGA NPs	anti-miR10b anti-miR 21	Breast cancer	PLGA-b-PEG	100-200 nm	[91]
	miR-34a	Breast cancer	PLGA-PLL	122 nm	[85]
	miR-34a	Multiple myeloma	PLGA-Chitosan	160 nm	[110]
	miR-150	Pancreatic cancer	PLGA	183 nm	[92]
Dendrimers	miR-194-5p miR-214-3p anti-miR-122-5p	Myocardial Infarction	PAMAM-His	60 nm	[99]
	anti-miR-21	Glioblastoma	PAMAM	100 nm	[100]

Abbreviations: PEI: Polyethyleneimine; PLL: poly L-Lysine; PLGA: polylactic-*co*-glycolic acid; PEG: polyethylene glycol; PAMAM: polyamidoamine; His: Histidine; N/A: not applicable.

## 3. Challenges and Perspectives in miRNA Delivery 

Currently, the inefficient miRNA delivery is the main drawback preventing their application in clinical routine. Uncomplexed miRNAs are unstable and would be rapidly degraded after entering the systemic circulation before reaching targeted tissue. Among the many challenges involved with in vivo miRNA delivery, the development of safe and efficient carriers remains the most prominent.

The clinical transition of miRNA therapeutics depends hence on the design of a suitable delivery system that can protect the small RNA molecules from nuclease degradation and promote their delivery into target tissues/cells, without inducing adverse effects [19].

As reported above, a wide range of vehicles has been explored for developing miRNA delivery systems. Suitable carriers must be non-toxic and non-immunogenic, provide serum stability and enhance miRNA cellular uptake in the target cells. Once inside cells, they should be able to release miRNA efficiently to trigger a biological response [119]. The choice of the appropriate carrier is influenced by the route of administration and therapeutic purposes (Table 4) [41].

**Table 4:** Examples of delivery methods used for in vivo miRNA delivery.

Delivery Method	miRNA	Disease	Route of Administration	Disease Model	Refs.
*Inorganic NPs*					
Gold Iron Oxide NPs	anti-miR-21 miR-100	Glioblastoma	Intranasal	U87-MG GBM cell-derived orthotopic mice xenograft	[47]
GD2 antibody targeted coated silica NPs	miR-34a	Neuroblastoma	Intravenous	NB1691^luc^ and SK-N-AS^luc^ orthotopic xenograft	[44]
*Lipid-based NPs*					
LNPs (DODMA/DOTAP/DOPC/CHOL/mPEG-DPPE)	anti-miR-21	Lung cancer	Intravenous	A549 mouse xenograft	[65]
SLNs (DDBA/Glyceryl monostearate/Soy phosphatidylcho-line/CHOL)	miR-34a	Lung cancer	Intravenous	in situ murine lung metastasis	[70]
*Polymeric NPs*					
uPAR targeted PLGA NPs	anti-miR-10b anti-miR-21	Breast cancer	Intravenous	TNBC xenograft	[91]
HA-CS targeted NPs	miR-34a	Breast cancer	Intratumoral	MDA-MB-231 mice xenograft	[113]

Abbreviations: GD2: disialoganglioside; uPAR: urokinase plasminogen activator receptor; PLGA: polylactic-co-glycolic acid; HA: Hyaluronic acid; CS: chitosan; GBM: glioblastoma multiforme; TNBC: triple negative breast cancer.

Among the different types of nanoparticles, LNPs, a relatively mature technology, are widely used for in vivo delivery of small interfering RNAs [57,58,62]. They are composed of a lipid bilayer that contains the miRNA encapsulated in the hydrophilic aqueous core or, complexed with the lipids on the surface [49,59]. The development of ionizable cationic lipids that are positively charged at acidic pH, but almost neutral at physiological pH, has proven to be essential for the development of clinically viable LNPs [57,120]. For example, the first siRNA-based therapy to be approved by the FDA, Onpattro^®^, is a LNP composed of heptatriaconta-6,9,28,31-tetraen-19-yl 4-(dimethylamino)butanoate (DLin-MC3-DMA) ionizable lipid [41,58].

Although there is still no FDA-approved miRNA therapeutics, some candidates have shown potential (e.g., Miravirsen, RG-101), while some have failed [33]. MRX34, a liposomal injection of miR-34 has been the first compound based on miRNA mimics to enter a phase I clinical trial to treat patients with advanced solid tumors. The trial was prematurely closed because of serious immune-related events which were potentially attributed to the liposome carrier instead of the miR-34 mimics themselves [53,121,122].

Cationic polymer-based systems have been widely investigated. The gold standard for the polymer-based gene delivery is PEI, which can bind to small RNAs to form nanosized complexes. Lin et al. have shown that branched PEIs were more effective at transferring miR-494 in mouse embryonic fibroblast cells than the lipofectamine reagent [123]. Despite being an efficient transfecting agent, it is highly cytotoxic.

The design of vehicles that improve miRNA delivery to specific tissues are being highly explored to achieve more desirable and on-target effects, enhancing the efficiency and specificity while decreasing side effects. Engineering nanoparticles with targeting moieties that are specific against cell surface receptors may increase cellular interactions. For example, in the work of Valcourt and Day, PLGA NPs functionalized with Notch1 antibodies were employed for the specific delivery of miR-34a into triple-negative breast cells (TNBC), an aggressive type of breast cancer, unsusceptible to current targeted or hormonal therapies. The overexpression of Notch1 receptors on the surface of TNBC was used as an attachment point for NPs binding, which facilitates their retention in the tumor microenvironment [124]. On the other side, Yin et al. constructed multifunctional NPs for the delivery of anti-miR-21 in TBNC incorporating an RNA aptamer against CD133, one of the best markers of cancer stem cells. An aptamer is a nucleic acid sequence with special three dimensional structure that has a high affinity to a chosen cell target [17]. The systemic injection of these RNA nanoparticles in animals demonstrated high specificity in TNBC tumor targeting and high efficacy for tumor growth inhibition thanks to an improved bioavailability [125].

In the work of Xue et al., they constructed a dendrimer NP based on PEGylated dendrigraft poly-L-lysine (PEG-DGL) anchored with angiotensin II type 1 (AT1) targeting peptide (AT1-PEG-DGL) to specifically deliver microRNA-1 inhibitor (AMO-1) to the ischemic cardiac tissues. The angiotensin II type 1 receptor (AT1R) was found to be over-expressed in the early stage of the ischemic heart. In vivo experiments after intravenous administration demonstrated quickly accumulation of AT1-PEG-DGL NPs when compared to the control group without AT1 targeting. The myocardial infarction (MI) size was reduced by 64.1% as compared with that in MI control group, very promising for early MI treatment [126].

**Figure 2:**
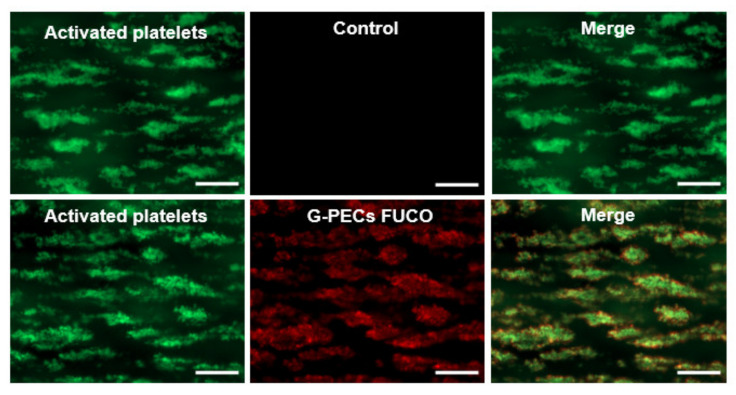
P-selecting targeting polysaccharide-based nanogels (G-PECs fuco) for specific miRNA delivery in atherothrombotic-related diseases. Particles and platelets aggregates co-localization was evaluated by merged fluorescence microscopy images (right). G-PECs without fucoidan were used as control. Scale bars corresponds to 50 µm. Reproduced with permission from Elsevier [127].

Besides the use of antibodies and aptamers, the use of natural compounds is promising because they might contribute to reduce the immunogenicity and toxicity of miRNA carriers. In our recent works, we have designed and synthetized biospecific polysaccharide NPs for the delivery of miRNA-based therapeutics in atherothrombotic-related diseases [127]. For this purpose, fucoidan, a naturally occurring sulfated polysaccharide extracted from brown algae, was employed as targeted moiety. Nanosystems decorated with fucoidan showed to be able to accumulate on activated platelets (Figure 2) thanks to the high affinity of fucoidan for P-selectin, an adhesion molecule over-expressed at the surface of activated platelets and endothelial cells. The ability of our nanosystems to target P-selectin make them attractive candidates for the delivery of small sized nucleic acids, while focused on the cardiovascular field.

After internalization by targeted cells, carriers need also to deal with nucleic acid fate. Successful use of any delivery vehicle requires payload release in the proper subcellular compartment [128,129]. The site of action differs according to the type of therapeutic nucleic acid: pDNAs should be delivered in the cell nucleus, whereas mRNA, siRNA or miRNA therapeutics have they target located in the cytosol [35]. Of note, in the study of Wittrup et al., they calculated the cytoplasmic delivery efficiency of small interfering RNAs around only 3.5% of the total nucleic acid internalized, highlighting the challenges concerning the cytoplasmic delivery of nucleic acids payloads from NPs [130].

Carriers normally are internalized by cells via endocytic pathways, and then nucleic acid is transferred from endosomes towards cytosol [131]. To avoid nucleic acid degradation, the nanocarrier should preferably escape from the endocytic vesicles into the cell cytoplasm and promptly release the payload in order to exert its therapeutic action [132]. For this purpose, “smart” materials (inorganic, lipids and polymers) can be engineered in order to release their payload upon different stimuli (e.g., temperature, pH, biological signals) [1,133,134]. A judiciously rational designed gene delivery system must be elaborated to have sufficient in vivo stability during circulation time of nucleic acid until it reaches targeted cells while being capable of rapid release after being taken up in order to maximize their therapeutic effect [119].

Finally, another greatest challenge in miRNA therapy is the evaluation of the changes in the target after miRNA delivery. When designing miRNA-based therapeutics, quantitative assays are necessary to check the amount of delivered miRNA and the effects on the protein production. Traditional approaches to assess miRNA silencing activity include the analysis of mRNA products such as relative quantity of the relevant RNA by real-time quantitative polymerase chain reaction (qPCR) or the expression of the encoded protein as measured by Western blot (WB) [135,136]. However, those techniques are labor intensive and time-consuming. In addition, they also require cell lysis, which make them unsuitable for dynamic analysis of expression level and/or in vivo detection of miRNAs.

Recent approaches on the analysis of the extent of gene silencing efficiency were done by using RNA interference (RNAi) against a reporter gene such as luciferase [137,138,139] or green fluorescence protein (GFP) [131,140,141]. Culture cells must stably express the reporter gene or be transfected first with a plasmid containing the reporter gene and then with the NP containing the RNAi of interest [142,143]. According to the design of the imaging system, an increase or reduction in the light emitted by cells is generated and can be monitored through optical imaging [135].

**Figure 3:**
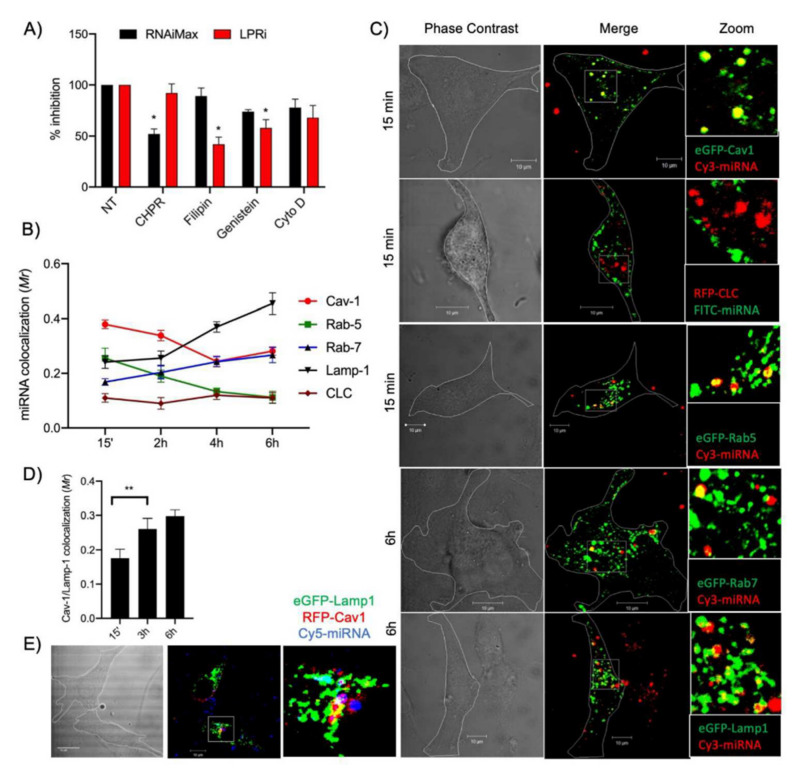
Intracellular trafficking of miRNA-133a. (**A**) U87MG LentiRILES/133 T cells were pre-incubated with specific inhibitors of the caveolae (Fillipin and Genistein), clathrin (Chlorpromazine, CHPR) and macropinocytosis (cytochalasin D, Cyto D) internalization pathways before miRNA-133a transfection with LPRi or RNAiMax. Then, 48 h later, the luciferase activity in cells was quantified (*n* = 3). (**B**) U87MG cells expressing eGFP-Cav1, RFP-CLC, eGFP-Rab5, eGFP-Rab7 or eGFPLamp1 were transfected for 1 h (pulse) with LPRi.Cy3-miRNA-133a then washed and further incubated for 15 min, 2, 4 or 6 h, fixed in 3% PFA and analyzed by confocal microscopy analysis (*n* = 4). (**C**) Representative images from eGFP-Cav1, RFP-CLC and eGFP-Rab5 cells collected at a 15-min time point and from eGFP-Rab7 or eGFP-Lamp1 cells collected at a 6 h time points. (**D**) Quantification of co-localization events between RFP-Cav1 and eGFP-Lamp1 detected at 15 min, 3 and 6 h postincubation (*n* = 3). (**E**) Co-localization of LPRi.Cy5-miRNA-133a with RFP-Cav1 and eGFP-Lamp1 after 3 h incubation (*n* = 3). For all panels, values are mean ± SEM; * *p* < 0.05, ** *p* < 0.01; Reproduced with permission from Elsevier [79].

Ezzine et al., have developed a miRNA-ON functional monitoring system called RILES, for RNAi-Inducible Luciferase Expression System that relies on an engineered regulatable expression system to switch-ON the luciferase/GFP expression gene when a miRNA of interest is functional inside the cells [144]. This positive monitoring system has been combined with confocal microscopy intracellular studies to delineate miRNA lipopolyplexes internalization pathway and processing by the RISC machinery in glioblastoma cells. In this study, cells have been transduced with lentiviral vectors expressing RILES. The results demonstrated that miRNA histidylated lipopolyplexes are preferentially uptaken through the caveolae-dependent endocytosis pathway and routed to the endosomal vesicles and multivesicular bodies (Figure 3). Interestingly, a part of miRNA oligonucleotides was caught by Argonaute 2 and trafficked to P-bodies for storage while some miRNAs were exocytosed through exosomes before being re-captured by the cells [79].

Turk et al. developed a GFP-based reporter system to monitor the cellular levels of the tumor suppressor miRNA let-7 in single Human embryonic kidney 293 (HEK 293) cells. Using qPCR, they confirmed the inverse relationship of GFP fluorescence and miRNA levels [145]. However, major drawbacks of reporter gene-based techniques include genetic modification of the studied objects, high tissue auto-fluorescence background (especially for fluorescence-based miRNA imaging) and poor tissue penetration of the light in vivo, which limit their use as a research tool in cells and small animal models.

The development of alternatives imaging modalities to monitor miRNA delivery and its therapeutic efficacy is of great interest. Besides the applications of optical imaging in miRNA research, other imaging modalities have been explored. For example, Yang et al. developed a poly(amidoamine) dendrimer-grafted gadolinium-functionalized nanographene oxide (Gd-NGO) nanoparticles for combined miRNA delivery and MRI. Together with the use of fluorescein amidite (FAM) labeled miRNA, these systems allowed the quantification of miRNA delivery in real time in vivo [54].

More recently, some studies about miRNA in vivo imaging have used the scintigraphy [146,147]. In the work of Simion et al., they combined the use of human sodium iodide symporter (hNIS) reporter gene and a single-photon emission computed tomography/computed tomography (SPECT/CT) camera to dynamically monitor the expression of miRNAs. They demonstrated a correlation between radiotracer (^99m^Tc) accumulation in transfected cells with the induction of hNIS and with the expression of miRNAs detected by real time PCR providing evidence that radionuclide imaging of miRNA expression using hNIS has potential for miRNA monitoring in clinical settings [148].

For a while, none of the methods discussed above are able to fulfill all the requirements necessary for miRNA monitoring. Hence, the combination of several molecular imaging modalities is of great value to offer complementary information about miRNA functions. In addition, the use of NPs as imaging agents can facilitate multimodal imaging due to their ability to incorporate different imaging markers in a single imaging compound thus enabling image-guided treatment approaches (theranostics).

## 4. Conclusions

The success of miRNA-based therapeutics depends on the development of clinically relevant delivery vectors able to avoid premature degradation of genetic material in the organism until reaching the final target. Despite significant progress in the field of non-viral vehicles, the discovery of an ideal delivery vector is still far away to be accomplished. Understanding the limitations and demands of current carriers together with tracking miRNA is essential for the coherent development of new ones [38,89,110]. Existing data are very promising and reinforce the utility of nanoparticles as important tools for efficient and controlled miRNA delivery. The recent approval of the first messenger RNA-based vaccine against COVID-19 pandemic and ongoing clinical trials with therapeutic nucleic acids highlight the feasibility of their use as therapeutics providing ground for the translation of more RNA-based therapeutics as drug products pipelines.

## Data Availability

Not applicable.

## Abbreviations

miRNA	MicroRNA
pDNA	Plasmid DNA
siRNA	Small interfering RNA
mRNA	Messenger RNA
RNAi	RNA interference
FDA	Food and drug administration
NPs	Nanoparticles
LNPs	Lipid-based nanoparticles
SLNs	Solid lipid nanoparticles
LPP	Lipopolyplexes
